# High homocysteine induces betaine depletion

**DOI:** 10.1042/BSR20150094

**Published:** 2015-07-07

**Authors:** Apolline Imbard, Jean-François Benoist, Ruben Esse, Sapna Gupta, Sophie Lebon, An S de Vriese, Helene Ogier de Baulny, Warren Kruger, Manuel Schiff, Henk J. Blom

**Affiliations:** * Biochemistry Hormonology Laboratory, Robert-Debré Hospital, APHP, 48 Bd Serurier, Paris 75019, France; † Paris Sud University, Pharmacy Faculty, 5 rue Jean Baptiste Clément, Chatenay-Malabry 92019, France; ‡ Institute for Medicines and Pharmaceutical Sciences (iMed.UL), Faculty of Pharmacy, University of Lisbon, Lisbon 1649-003, Portugal; § Cancer Biology Program, Fox Chase Cancer Center, Philadelphia, PA 19111, U.S.A.; ║ INSERM UMR 1141, Hôpital Robert Debré, 48 Bd Serurier, Paris 75019, France; ¶ Renal Unit, Department of Internal Medicine, AZ Sint-Jan AV, Bruges 8000, Belgium; * *Reference Center for Inherited Metabolic Diseases, Robert-Debré Hospital, APHP, Paris 75019, France; † †Faculté de Médecine Denis Diderot, Université Paris Diderot–Paris 7, Paris 75013, France; ‡ ‡Laboratory for Clinical Biochemistry and Metabolism, Department of General Pediatrics, Center for Pediatrics and Adolescent Medicine University Hospital, Freiburg D-79106, Germany

**Keywords:** betaine, cystathionine-β-synthase deficiency, hyperhomocysteinaemia, S-adenosyl-methionine

## Abstract

Betaine is the substrate of the liver- and kidney-specific betaine-homocysteine (Hcy) methyltransferase (BHMT), an alternate pathway for Hcy remethylation. We hypothesized that BHMT is a major pathway for homocysteine removal in cases of hyperhomocysteinaemia (HHcy). Therefore, we measured betaine in plasma and tissues from patients and animal models of HHcy of genetic and acquired cause. Plasma was collected from patients presenting HHcy without any Hcy interfering treatment. Plasma and tissues were collected from rat models of HHcy induced by diet and from a mouse model of cystathionine β-synthase (CBS) deficiency. S-adenosyl-methionine (AdoMet), S-adenosyl-homocysteine (AdoHcy), methionine, betaine and dimethylglycine (DMG) were quantified by ESI—LC–MS/MS. mRNA expression was quantified using quantitative real-time (QRT)-PCR. For all patients with diverse causes of HHcy, plasma betaine concentrations were below the normal values of our laboratory. In the diet-induced HHcy rat model, betaine was decreased in all tissues analysed (liver, brain, heart). In the mouse CBS deficiency model, betaine was decreased in plasma, liver, heart and brain, but was conserved in kidney. Surprisingly, *BHMT* expression and activity was decreased in liver. However, in kidney, *BHMT* and *SLC6A12* expression was increased in CBS-deficient mice. Chronic HHcy, irrespective of its cause, induces betaine depletion in plasma and tissues (liver, brain and heart), indicating a global decrease in the body betaine pool. In kidney, betaine concentrations were not affected, possibly due to overexpression of the betaine transporter *SLC6A12* where betaine may be conserved because of its crucial role as an osmolyte.

## Abstract (precis)

We demonstrated in the present study that betaine-homocysteine (Hcy) methyltransferase (BHMT) is a major pathway for Hcy removal in all situations of hyperhomocysteinaemia (HHcy). Hperhomocysteinaemia induces betaine depletion in plasma and tissues except in kidney, where betaine may play a crucial role as an osmolyte.

## INTRODUCTION

Homocysteine (Hcy) is a toxic compound involved in the pathophysiology of various disorders [1]. It is produced in the methylation cycle, an essential cellular process, including DNA methylation, protein methylation, lipids synthesis, neurotransmitters synthesis and detoxification through donation of the methyl group of S-adenosyl-methionine (AdoMet), also called the universal methyl donor (Figure 1) [2]. Upon transfer of the methyl group, AdoMet is converted into S-adenosyl-homocysteine (AdoHcy), which is hydrolysed by AdoHcy hydrolase to adenosine and Hcy. Hcy cellular concentrations are tightly regulated. Hcy can be either catabolized trough the *trans*-sulfuration pathway or remethylated into methionine. This remethylation into methionine can occur via a ubiquitous pathway catalysed by methionine synthase (MS), which uses 5-methyltetrahydrofolate as methyl donor or via the alternative tissue-specific betaine-homocysteine methyltransferase (BHMT) using betaine as methyl donor. BHMT is mainly expressed in liver and kidney. However, despite this limited expression, BHMT converts a major part of Hcy formed in the body [3–5].

**Figure 1:**
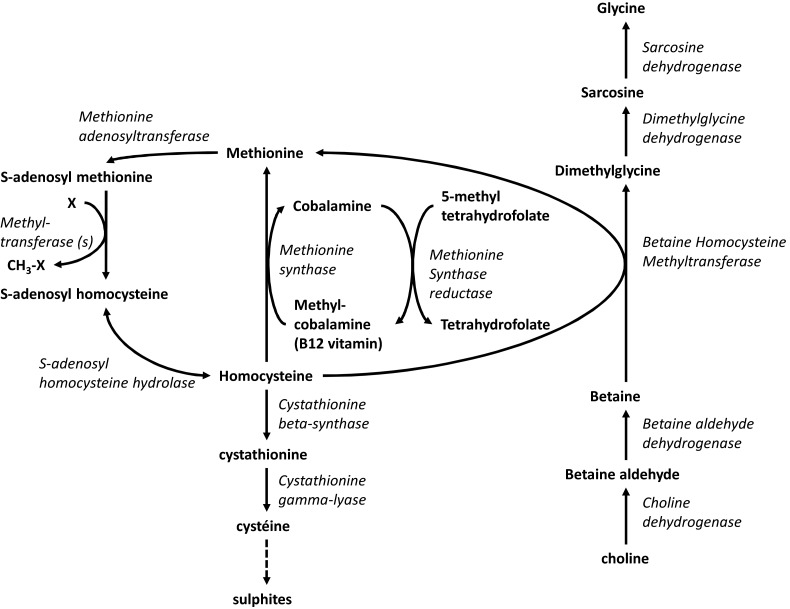
Metabolism of Hcy remethylation and trans-sulfuration and its iterconnexion with metabolism of betaine Enzymes are presented in italic

Betaine has for long been used as a component of the therapeutic strategy, together with dietary restriction and vitamin supplementation, in classical homocystinuria [cystathionine β-synthase (CBS) deficiency] in order to lower Hcy concentrations [6]. Betaine therapy alone has been shown to reduce Hcy concentrations and to prevent vascular complications, among others [7]. Betaine has also been extensively used in remethylation disorders [e.g. intracellular cobalamin metabolism and methylene-tetrahydrofolate reductase (MTHFR) deficiencies] to lower Hcy concentrations and to increase methionine concentrations [8].

Betaine can either be provided by the diet or produced *in vivo* from its precursor choline, also supplied by diet [9]. Betaine is abundant in cereals, especially wheat and in vegetables of the beet family, including spinach, chard and beetroot [9]. There is a general agreement that betaine dietary supply is between 100 and 300 mg/day [10]. The conversion of choline to betaine is a two-step pathway localized in the liver and kidney mitochondria [10] (Figure 1). There are no quantitative estimates of the relative importance of *in vivo* synthesis compared with food intake, but the importance of these sources could be expected to vary with dietary habits. Dietary choline could, in principle, supply all the requirements for both choline and betaine. The converse does not follow, although betaine does have a choline-sparing effect [11]. Betaine is very well reabsorbed in the kidney and the majority of its elimination is through its metabolization by BHMT.

Beside its role as methyl-donor for Hcy remethylation, betaine plays other essential functions in mammalian cells. It is a small zwitterionic compound at neutral pH that behaves like an essential osmolyte to maintain cellular volume under osmotic stress [10]. This function is particularly important in the kidney medulla, where betaine is present at higher concentrations than in other tissues [12]. Betaine is also a ‘chemical chaperone’, providing protection against protein denaturation, which may contribute to its therapeutic effect in patients with homocystinuria [13].

We hypothesized that BHMT is a major pathway for Hcy removal specifically in cases of hyperhomocysteinaemia (HHcy). The aim of this study was therefore to evaluate the betaine status in patients and animal models with HHcy of diverse causes before any treatment.

## EXPERIMENTAL

### Patients

#### Inclusion criteria

We retrospectively analysed the biological results of patients with various forms of HHcy of identified cause before any treatment, for whom total plasma Hcy (tHcy), methionine, betaine and dimethylglycine (DMG) were measured in our laboratory between 2011 and 2013 in the context of the standard patient's management.

#### Plasma sampling

Blood was drawn in lithium heparinate and centrifuged 10 min at 4°C, 3380 ***g***. Plasma was aliquoted and stored at −20°C until measurement (within 1 month after drawing).

### Animals

#### Nutritionally induced HHcy model in rats

The study was performed in female Wistar rats with a body weight of approximately 250 g (Iffa Credo), in accordance with local prescriptions and the NIH Guide for the Care and Use of Laboratory Animals. During 8 weeks, animals were maintained on one of the following diets: standard rodent chow (controls); diet enriched in methionine (HM); diet deficient in folate, vitamin B6 and vitamin B12 (folate: 0.08 mg/kg, vitamin B6: 0.01 mg/kg, vitamin B12: 10.4 μg/kg; LV); diet enriched in methionine and deficient in folate, vitamin B6 and vitamin B12 (HMLV) [14]. Then, the animals were anaesthetized with thiobutabarbital [Inactin, RBI; 100 mg/kg intraperitoneally (IP)] and blood was collected by cardiac puncture. Samples of liver, heart and brain were cut into small pieces using a surgical blade, weighed and stored at −80°C until use.

#### CBS mouse model

We used a previously described mouse model of CBS deficiency [8]. Briefly, transgenic mice in the C57BL6 strain background were generated in which the gene construct harbours the human *CBS* with the mis-sense mutation 833 T>C. Its expression is under control of the metallothionein promoter and is stimulated by adding zinc to the drinking water, yielding the mutant I278T human CBS. These animals, termed Tg-I278T *Cbs*^+/+^, were then mated to *Cbs*^+/−^ heterozygotes to obtain Tg-I278T *Cbs*^+/−^ offspring. Tg-I278T *Cbs*^+/−^ mice were then put on water containing 25 mM zinc sulfate to induce transgene expression and backcrossed a second time to *Cbs*^+/−^ animals to generate Tg-I278T *Cbs*^−/−^ mice. The residual activity of the mutant human isoform rescues the neonatal lethality phenotype of *Cbs*^−/−^. After weaning, zinc water was replaced by plain water. The animals were fed standard rodent chow (Teklad 2018SX; Harlan Teklad) *ad libitum* and killed after 50–330 days of birth. The brain, liver, heart and kidney were then extracted and stored at −80°C until further analysis. These studies were approved by the Fox Chase Cancer Center IACUC.

#### Tissues preparation

Tissues homogenate were prepared on ice in 0.1 N HCl (10 ml/g wet weight of tissue) with an OMNI-2000 homogenizer (Omni 2000, OMNI International). Immediately after homogenization, a part of the homogenate was processed for AdoMet and AdoHcy determination, as described below. The remainder was aliquoted and stored at −80°C until use.

### Metabolites measurement

For quantification of AdoMet and AdoHcy, the tissue homogenate was promptly diluted 10-fold in 0.1 N HCl under ice to a final volume of 250 μl, followed by addition of 156 μl of 10% perchloric acid for deproteinization. After centrifugation for 10 min at 2000 ***g*** at 4°C, the supernatant was subjected to a weak anion-exchange solid-phase extraction procedure using a Waters Oasis HLB 3cc (30 mg) extraction column (Waters) and analysed by stable-isotope dilution LC–MS/MS, as previously described in detail [15].

tHcy and methionine concentrations were measured by a previously described ESI–LC–MS/MS method [16].

Betaine and DMG were simultaneously assayed (in plasma and tissue homogenates) after deproteinization by a previously described LC–MS/MS method [17].

All metabolites measurements performed in tissue homogenates were normalized with protein content. Protein concentration was determined with the Bicinchoninic Acid Protein Assay Kit (Pierce) using BSA as the standard.

### BHMT activity

Approximately 20 mg of tissue was ground in cold potters in 1 ml of 50 mM KH_2_PO_4_ buffer, pH 8. Seven-hundred microlitres of the homogenate were mixed with 100 μl of 35 mM DTT and 100 μL of Hcy 10 mM and pre-incubated 5 min at 37°C. The reaction was then started by adding 100 μl of 20 mM betaine. Every 10 min up to 40 min, 50 μl of the reaction mixture was sampled and added to 450 μl of methanol to stop the reaction. After centrifugation, the supernatant were transferred to a new eppendorf with 5 μl of DTT 500 mM and 10 μl of methionine-d3 500 μM. Each sample was dried under a stream of nitrogen and derivatized using 200 μl of HCl/Butanol during 15 min at 60°C. Finally, samples were dried under a stream of nitrogen and solubilized in 100 μl of 200 mM, pH 7.4, phosphate buffer. Methionine and methionine-d3 were measured in each sample by ESI–LC–MS/MS in the multiple reaction monitoring mode by a previously described LC–MS/MS method [17]. The results were expressed in percentage compared with the mean of the controls (Tg-I278T *Cbs* ^−/−^) measured in the same batch.

### Quantitative PCR

Total RNA of hepatic and renal tissues was extracted from 5 to 10 mg of tissue using the RNeasy Lipid Tissue Mini Protocol Kit from Qiagen according to the manufacturer instructions (Qiagen). The obtained RNA was quantified using a Nanodrop 2000 (Thermo scientific). Real-time (RT)-PCR was performed using 1 μg of RNA with the iScript cDNA Synthesis Kit according to the manufacturer instructions (Biorad Laboratories). Newly primers were designed for choline dehydrogenase (*CHDH*), betaine aldehyde dehydrogenase (*BADH*), *BHMT*, dimethylglycine dehydrogenase (*DMGDH*), sarcosine dehydrogenase (*SARDH*), *SLC6A12* (primers upon request). Quantitative (Q)-PCR was performed on a thermocycler CFX384 Biorad C1000 (annealing temperature 60°C and 40 cycles) with SYBRgreen as fluorophore. A melting curve between 65°C–95°C was performed to verify the quality of the amplification. Each sample was quantified in duplicate. *RPL13* was used as reference gene.

### Statistics

Differences between several groups were first sought by the Kruskal–Wallis test. If the test showed significant differences (*P*<0.05), groups were tested in pairs by the Mann–Whitney test. Statistical analyses were performed using SPSS 17.0 for Windows (SPSS Inc.) and GraphPad Prism 5.0 (GraphPad Software).

### Study approval

Experiments on rat were performed in accordance with the NIH Guide for the Care and Use of Laboratory Animals. Experiments on the CBs mouse model were approved by the Fox Chase Cancer Center IACUC.

## RESULTS

### Patients

In total, we included 17 patients, of which eight had a genetic cause of HHcy: three patients with classical homocystinuria (CBS deficiency), four patients with cblC deficiency and one patient with MTHFR deficiency. Five patients had acquired HHcy: three breast-fed babies from mothers with acquired Biermer anaemia, one adolescent with a B12-deficient diet and one patient with short bowel syndrome. One patient had HHcy due to excessive consumption of nitrous oxide. Finally, one patient had HHcy induced by a folate-deficient diet and two patients had HHcy due to methotrexate treatment.

### Concentrations of plasma metabolites in patients

tHcy, methionine, betaine and DMG concentrations of patients are presented Table 1. Betaine concentrations were below the normal ranges of our laboratory (Figure 2) for all patients, whatever the aetiology of HHcy.

**Figure 2:**
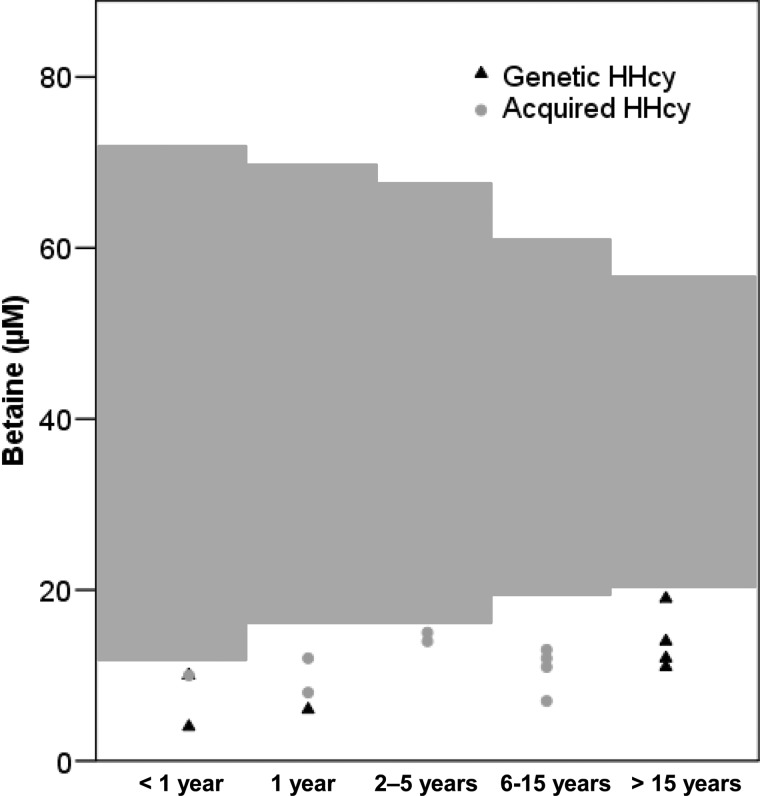
Plasma concentrations of betaine in patients with HHcy Grey areas represent reference range of betaine concentrations according to age.

**Table 1:** Plasma biochemical parameters of patients with HHcy

	Genetic HHcy								Acquired HHcy								
Pathology	Classical homocystinuria			Cblc def.				MTHFR def.	B12 def.					N_2_O ttt	Folate def.	Methotrexate ttt	
Patient N°	1	2	3	4	5	6	7	8	9	10	11	12	13	14	15	16	17
Age at sampling	17 Y	26 Y	31 Y	14 D	24 D	3 M	18 Y	1 D	4 M	6 M	15 Y	11 M	11Y	12 Y	4 Y	14 Y	4 Y
tHcy (μM)	262*	631*	322*	127*	211*	154*	89*	155*	124*	90*	122*	125*	111*	155*	59*	40*	31*
Met (μM)	626*	322*	593*	2^†^	5^†^	7^†^	32	3^†^	7^†^	5^†^	20	9^†^	19^†^	11^†^	16^†^	11^†^	26
Betaine (μM)	11^†^	19^†^	12^†^	10^†^	10^†^	6^†^	14^†^	4^†^	8^†^	12^†^	7^†^	10^†^	13^†^	11^†^	14^†^	12^†^	15^†^
DMG (μM)	4.0	2.4	3.9	4.0	11.8	3.0	7.0	1.0^†^	8.0	9.0	22.2*	6.0	1.6	1.3	3.6	4.1	1.9

*Concentration above the laboratory reference values according to age.

^†^ Concentration below the laboratory reference values according to age.

D, days, def., deficiency; M, months; Met, methionine; N_2_O, nitrous oxide; tHcy, total homocysteine; ttt, treatment; Y, years.

Patients with acquired HHcy were treated with vitamin therapy without betaine treatment. For those patients, evolution of betaine and tHcy concentrations during the follow-up after treatment is showed in Table 2 when available. Normalization of tHcy and betaine concentrations were observed at the first control sample (day 6) after B12-vitamin treatment for patients 9 and 12. Betaine normalization was delayed in time compared with tHcy in patients 10 and 11 (at day 10 and 16 respectively). tHcy concentrations of patient 14 reached tardily normal range because of the persistence of nitrous oxide administration, but betaine was normal at the first sample collected after treatment (at day 24).

**Table 2:** Evolution of plasma tHcy and betaine in patients with acquired HHcy after treatment with B12 vitamin

Patient number	Pathology			Treatment ↓											
9	B12 deficiency	Time after treatment (days)	0			6	9		13						
		tHcy (μM)	124			6	5		5						
		Betaine (μM)	8			38	25		28						
10	B12 deficiency	Time after treatment (days)	0	4		6		10							
		tHcy (μM)	90	10		3		3							
		Betaine (μM)	12	17		15		49							
11	B12 deficiency	Time after treatment (days)	0		5					16					
		tHcy (μM)	122		8					6					
		Betaine (μM)	7		8					30					
12	B12 deficiency	Time after treatment (days)	0			6							60		
		tHcy (μM)	125			6							6		
		Betaine (μM)	10			30							28		
13	B12 deficiency	Time after treatment (days)	0		5										
		tHcy (μM)	111		67										
		Betaine (μM)	13		14										
14	N_2_O-induced HHcy	Time after treatment (days)	0									24		68	80
		tHcy (μM)	155									66		23	6
		Betaine (μM)	11									75		57	82
15	Folate deficiency	Time after treatment (days)	0	ND											
		tHcy (μM)	59												
		Betaine (μM)	14												
16	Methotrexatetreatment	Time after treatment (days)	0	ND											
		tHcy (μM)	40												
		Betaine (μM)	12												
17	Methotrexate treatment	Time after treatment (days)	0	ND											
		tHcy (μM)	31												
		Betaine (μM)	15												

N_2_O, nitrous oxide

ND, no data

### Concentrations of plasma metabolites in animal models

#### Rat nutritional model of HHcy

In rats with nutritional HHcy, all groups had significantly increased tHcy concentrations compared with controls (Figure 3) and rats of HMLV group had significantly higher tHcy concentrations than HM and LV groups (*P*<0.001 and *P*=0.004 respectively). Betaine concentrations were decreased in HMLV and HM groups, but not in the LV group, compared with controls.

**Figure 3:**
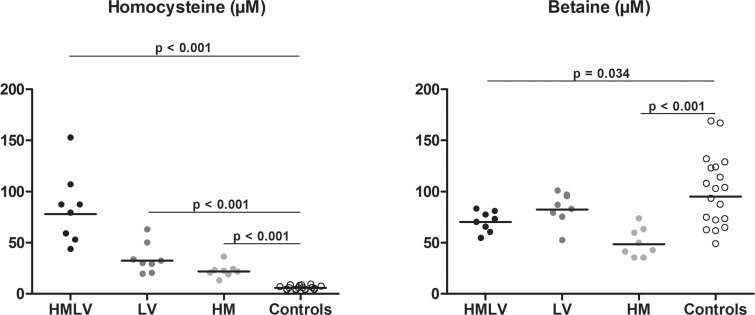
Plasma concentrations of tHcy and betaine in rats nutritional models of HHcy HM: High methionine diet; HMLV: High methionine low B vitamin diet (B6, B9, B12); LV: Low vitamin diet (B6, B9, B12); Controls: standard rodent diet; Comparisons were made using the Mann–Whitney test between controls and each group of diet induced HHcy (HMLV, HM and LV respectively). Only the significant comparisons (*P*<0.05) using the Mann–Whitney test are represented in the figures.

#### Mouse model of CBS deficiency

In the mouse model of CBS deficiency, Tg I278T *Cbs*^−/−^ mice had a dramatically increased plasma tHcy concentrations compared with Tg I278T *Cbs*^+/+^ (Figure 4). Tg I278T *Cbs*^+/−^ mice had a milder increased tHcy concentration. Plasma methionine was not different between the three groups (result not shown). Betaine concentrations were significantly decreased in Tg I278T *Cbs*^−/−^ mice and a non-significant tendency was observed in the Tg I278T *Cbs*^+/−^ compared with Tg I278T *Cbs*^+/+^.

**Figure 4:**
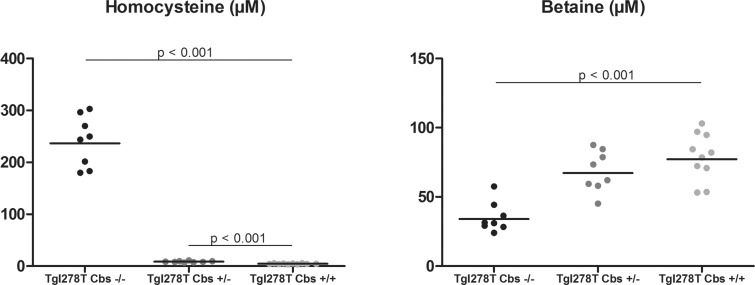
Plasma concentrations of tHcy and betaine in the mouse model of CBS deficiency Comparisons were made using the Mann–Whitney test between control mice Tg I278T Cbs^+/+^ and homozygous deficient mice Tg I278T Cbs^+/^− or heterozygous deficient mice Tg I278T Cbs^−/−^. Only the significant comparisons (*P*<0.05) using the Mann–Whitney test are represented in the figures.

### Concentrations of tissue metabolites in animal models of hyperhomocysteinaemia

#### Rat nutritional model of HHcy

In liver homogenates, HMLV and LV groups showed decreased AdoMet/AdoHcy ratio compared with controls (Figure 5). This was not found in brain and heart. In liver, brain and heart homogenates, all groups of nutritional induced HHcy showed decreased betaine concentrations compared with controls (Figure 5).

**Figure 5:**
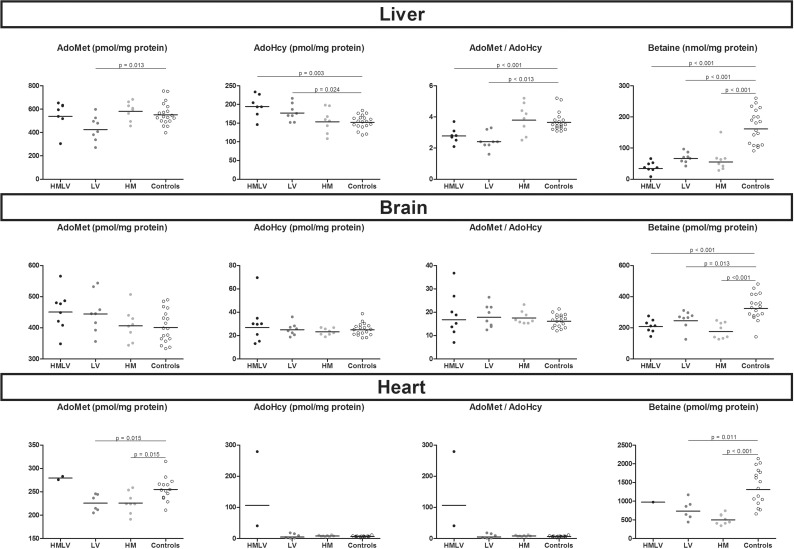
Concentrations of AdoMet, AdoHcy, AdoMet/AdoHcy ratio and betaine in tissues homogenates of liver, brain and heart in rats nutritional models of HHcy HM: High methionine diet; HMLV: High methionine low B vitamin diet (B6, B9, B12); LV: Low vitamin diet (B6, B9, B12); Controls: standard rodent diet; Comparisons were made using the Mann–Whitney test between controls and each group of diet induced HHcy (HMLV, HM and LV respectively). Only the significant comparisons (*P*<0.05) using the Mann–Whitney test are represented in the figures.

#### Mouse model of CBS deficiency

Tg I278T *Cbs*^−/−^ mice showed increased AdoMet concentrations in liver, brain and kidney homogenates and an increased AdoHcy in all tissues analysed compared with Tg I278T *Cbs*^+/+^ mice. In total, Tg I278T *Cbs*^−/−^ mice displayed significant decreased AdoMet/AdoHcy ratio in all tissue homogenates analysed (liver, brain, heart and kidney) compared with Tg I278T *Cbs*^−/−^ (Figure 6). Methionine was decreased in the liver homogenates of Tg I278T *Cbs*^−/−^ compared with Tg I278T *Cbs*^+/+^ (*P*=0.001, result not shown). Tg I278T *Cbs*^−/−^ mice presented betaine concentrations decreased in liver, brain and heart homogenates; also it was not significant in brain. This decreased was not found in kidney homogenates where betaine concentrations are the highest. In liver, Tg I278T *Cbs*^+/−^ showed intermediate betaine concentrations significantly different from both Tg I278T *Cbs*^−/−^ and Tg I278T *Cbs*^+/+^ mice.

**Figure 6:**
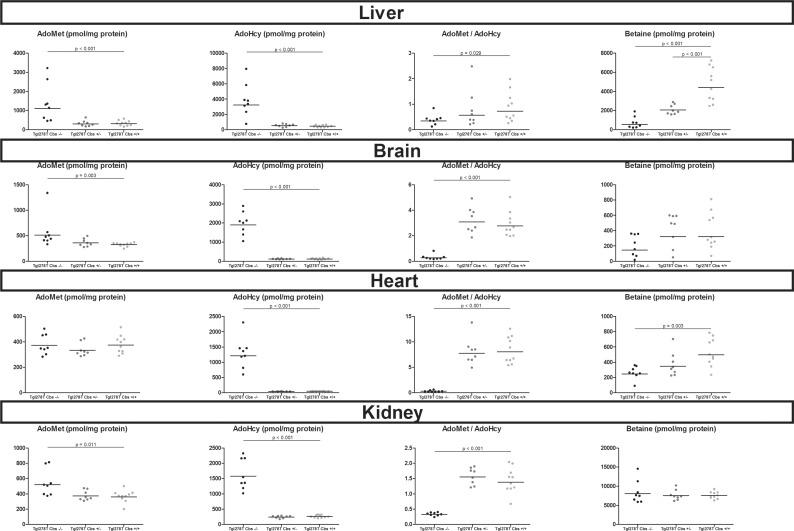
Concentrations of AdoMet, AdoHcy, AdoMet/AdoHcy ratio and in tissues homogenates of liver, brain and heart in the mouse model of CBS deficiency Comparisons were made using the Mann–Whitney test between control mice Tg I278T Cbs^+/+^ and homozygous deficient mice Tg I278T Cbs^+/−^ or heterozygous deficient mice Tg I278T Cbs^−/−^. Only the significant comparisons (*P*<0.05) using the Mann–Whitney test are represented in the figures.

### Tissues mRNA expression of gene of the betaine metabolism pathway in the CBS-deficient mouse model

We quantified the gene expression of the genes involved in betaine transport (cellular BGT1 transporter coded by the *SLC6A12* gene), synthesis (*CHDH* and *BADH*) and catabolism (*BHMT*, *DMGDH*, *SARDH*; Figure 7). In liver, expression of *BHMT* was decreased whereas it was increased in kidney of Tg I278T *Cbs*^−/−^ mice. In kidney, *DMGDH* and *BADH* expression was decreased, whereas *SLC6A12* expression was in increased in Tg I278T *Cbs*^−/−^ mice.

**Figure 7:**
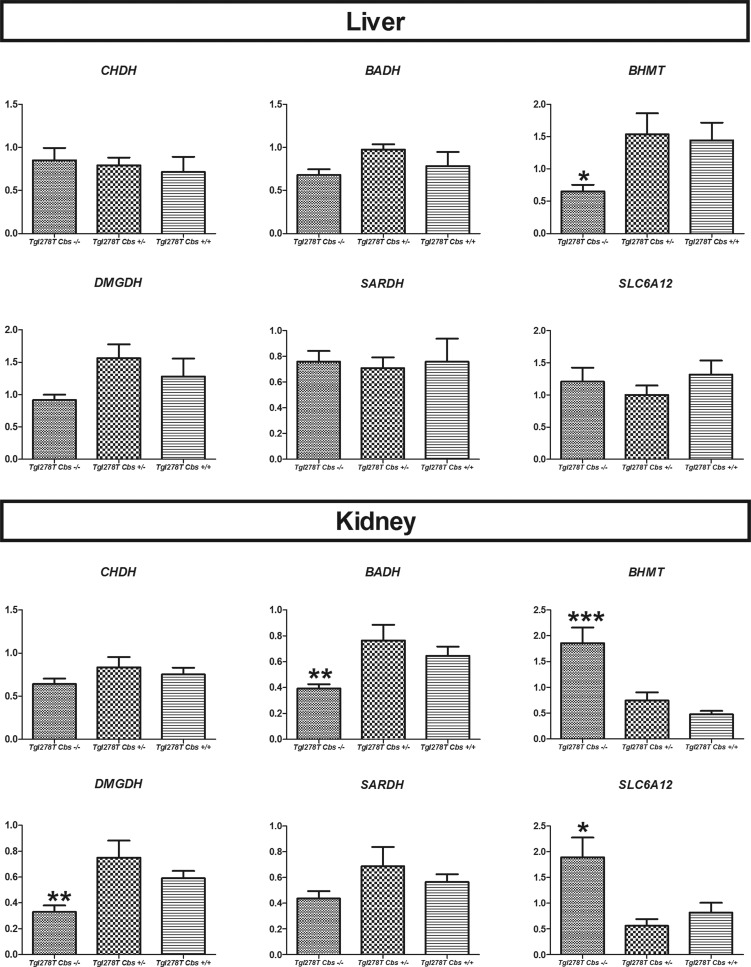
mRNA expression of the genes involved in betaine metabolism in liver and kidney homogenates in the mouse model of CBS deficiency Each sample was quantified in duplicate. RPL13 was used as reference gene. Comparisons were made using the Mann–Whitney test and only the significant comparisons were represented in the figure (*P*<0.05). *P<0.05, **P<0.01, ***P<0.001.

### BHMT activity in liver homogenates pathway of the CBS deficient mouse model

In liver homogenates, the decreased BHMT expression in the Tg I278T *Cbs*^−/−^ mice was accompanied by a decrease in BHMT activity (*P*=0.027; Figure 8). The increased *BHMT* expression in kidney homogenates of Tg I278T *Cbs*^−/−^ mice could not be confirmed at the activity level due to a very low basal BHMT activity in this tissue.

**Figure 8:**
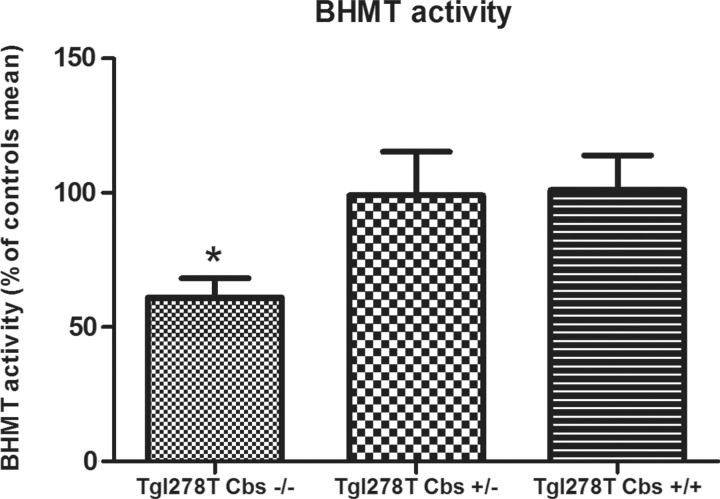
Enzyme BHMT activity in liver homogenates in the mouse model of CBS deficiency Only the significant comparisons using the Mann–Whitney test are presented in the figure. *P<0.05

## DISCUSSION

In the present study, we showed that betaine is depleted in patients and animals with HHcy, regardless of the aetiology of HHcy. In animal models, betaine was also decreased in liver, brain and heart, but not in the kidney. Only one previous study has reported decreased betaine concentrations in HHcy concerning plasma of three patients with classical homocystinuria [18].

Since we studied a paediatric population, we adapted our reference range according to age. We found a slight variation according to age during childhood (Figure 2), with a higher dispersion in younger patients. Our reference range for plasma betaine concentrations are comparable with those described in the literature [19–22]. Plasma betaine concentrations have been described to vary according to sex in adult patients [21] and to show very little intra-individual variations but large variation within the population, which explains the large reference range described [23]. In all patients with HHcy before any treatment, plasma betaine concentrations were decreased (Table 1; Figure 2). The decreases in betaine were quite substantial, on average 60% of the lower normal value. Decreased betaine concentrations in patients with remethylation defects (e.g. MTHFR, MS deficiency) may not be surprising because, in these patients, BHMT remains the only enzyme able to remethylate Hcy into methionine. Moreover, in all causes of HHcy, increased betaine consumption may occur because of the accumulation of Hcy, the BHMT substrate. However, there are very few data on betaine concentrations in patients with HHcy, since these diseases are relatively rare and betaine concentrations are thus far not evaluated in the management of these patients.

Patient 14 had HHcy induced by high doses of nitrous oxide. Nitrous oxide intoxication has been previously reported as a cause of sub-acute combined spinal cord degeneration associated with high concentrations of plasma tHcy [24,25]. It is believed that this effect is mediated by irreversible binding of nitrous oxide to the cobalt atom of B12-vitamin, the cofactor of MS, leading to oxidation of MS and so impaired remethylation of Hcy [26,27].

In patients with genetic causes of HHcy, betaine is generally part of the treatment. Therefore, we evaluated the effect of treatment on betaine and tHcy plasma concentrations only in patients with acquired HHcy when available (Table 2). For those patients, the treatment consisted of *per-os* or intra-muscular B_12_-vitamin. As soon as B_12_-vitamin is given, it will enable Hcy remethylation via MS. (Figure 1). This should allow to spare betaine. Indeed, plasma betaine concentrations normalized within 6–16 days, which indicates that dietary intake alone of betaine and its precursor choline was enough to correct betaine depletion.

We also studied plasma tHcy and betaine concentrations in two animal models of HHcy: a model of diet-induced HHcy in rats and a model of genetic cause of HHcy (CBS deficient mouse model). In rats, HHcy was induced by B-vitamin deficiency, methionine overload or a combination of both. In all cases, an increase in plasma tHcy was observed (Figure 3). In the nutritional models, there are large inter-individual variations, probably because all biological conditions cannot be controlled. In addition, the nutritional model is a less severe model of HHcy than the genetic mouse model of CBS deficiency; tHcy plasma concentrations in the nutritional model are increased between three and 13 times compared with controls and 60 times in the CBS-deficient mouse model. As in patients, in both animal models of HHcy we observed decreased betaine concentrations, at the exception of rats with HHcy induced with low B-vitamin diet, showing lower betaine levels but without statistical significant difference. Taken altogether, these data about plasma betaine concentrations suggest that all chronic situations of HHcy will cause decreased plasma betaine concentrations.

As it was not possible to investigate betaine tissue concentrations in patients, we measured tissue betaine concentrations in our two animal models of HHcy. We chose to measure betaine concentrations in liver, since it is the organ that has the highest expression of BHMT and in which it is one of the most abundant cytosolic proteins [28–30]. It has been previously shown that BHMT expression pattern was similar in rodents and in human [30]. Moreover, liver is considered to be the key organ for Hcy metabolism because all enzymes of betaine metabolism, remethylation cycle and trans-sulfuration are expressed in it [31]. In a previous study using an *in vitro* model of rat liver, it has been calculated that Hcy remethylation through BHMT or through MS is quantitatively equivalent and each processes ~25% of the cellular, Hcy whereas the trans-sulfuration pathway degrades approximately 50% of Hcy [32]. In both models, betaine concentrations were dramatically decreased in the liver homogenates in animals with HHcy. For the rat nutritional model, betaine was decrease in all HHcy diets, including in the LV group, which showed low-normal plasma betaine concentrations. In the CBS deficient mouse model, there was a decrease in betaine in the Tg I278T *Cbs*^−/−^ mice and also in heterozygous Tg I278T *Cbs*^+/−^ mice compared with Tg I278T *Cbs*^+/+^. This is supported by a previous study on an animal model of MTHFR deficiency reporting decreased liver betaine concentrations despite conserved plasma betaine concentrations [33].

Decreased betaine in liver could result from a higher consumption via BHMT enzyme or a decreased synthesis from choline or a decreased cellular transfer. To investigate this, we measured the expression of the genes involved in the metabolism of betaine model of CBS deficiency and the BHMT activity. Contrary to what we expected, expression and activity of BHMT was reduced in the Tg I278T *Cbs*^−/−^ mice liver (Figures 7 and 8). Similar results were observed by Mclean et al. [34] in a mouse CBS knockout model co-expressing at very low level the human transgene. In contrary, a previous study from Alberto et al. [35] showed an increased *BHMT* expression contrasting with a decreased BHMT activity in the liver of a CBS null mouse model. But in the latter model, Alberto and co-workers used a lethal complete knockout mouse model of CBS that requires choline supplementation to be viable. Choline, the precursor of betaine, has been shown to induce *BHMT* expression and activity [36]. In our mouse model, decrease in BHMT expression and activity might be induced via the increased AdoMet concentrations. AdoMet has been shown to be an inhibitor of *BHMT* expression [37,38]. We like to suggest that despite decreased BHMT expression and activity, the decrease in the hepatic betaine pool could be due to increased Hcy metabolism through BHMT. *In vivo* flux studies would be needed to confirm this hypothesis.

To further evaluate betaine pool, we measured betaine in brain and heart, two tissues where BHMT is not expressed [30]. In brain, betaine cellular transporter BGT1 is present, but basal betaine concentrations are very low and a potential function of betaine is not known [39]. In heart, betaine accumulation has been shown to be dependant only of plasma concentrations [40]. In our study, betaine concentrations were decreased in both animal models in brain and heart homogenates. BHMT is the only enzyme able to consume betaine and not expressed in these tissues. Our findings suggest an exchange of betaine between plasma and tissues.

In contrast with other tissues, betaine concentrations were not decreased in kidney of the Tg I278T *Cbs*^−/−^ mice, despite the presence of BHMT enzyme and increased HHcy concentrations in this tissue. It is noteworthy that betaine concentrations are the highest in kidney compared with the other tissues. In order to understand the preservation of betaine concentrations in kidney, we studied the expression of genes involved in the betaine metabolism pathway. We found an increased BHMT expression, which is unexpected regarding the conserved betaine concentrations and the increased concentrations of AdoMet, which supposedly inhibits *BHMT* expression (Figure 5). We also found an increased gene expression of *SLC6A12* coding for the cellular betaine transporter BGT1. According to Kempson et al. [39], the betaine concentration is tightly regulated in the kidney especially by BGT1 to counteract osmotic stress. In HHcy, the existence of osmotic stress in kidney has not been described. The increased expression of *SLC6A12* could explain the conserved betaine concentrations in this organ, although the mechanism of this up-regulation in HHcy is unknown.

In the present study, we showed that genetically as well as environmentally induced HHcy causes betaine depletion, at the exception of the kidney. Tissue storages of betaine could be redirected to the liver and the kidney to remethylate Hcy. In kidney, betaine concentrations are probably critical because they are stable, despite the global betaine depletion in other tissues. This might be due to the osmotic role of betaine, especially in the renal medulla. Because of the multiple roles of betaine as methyl-donor, osmolyte and chaperone molecule, betaine depletion could participate in the phenotype observed in patients with HHcy. It should be considered to treat patients with HHcy initially with betaine.

## Abbreviations

AdoHcy: S-adenosyl-homocysteine
AdoMet: S-adenosyl-methionine
BADH: betaine aldehyde dehydrogenase
BHMT: betaine-homocysteine methyltransferase
CBS: cystathionine β-synthase
CHDH: choline dehydrogenase
DMG: dimethylglycine
DMGDH: dimethylglycine dehydrogenase
Hcy: homocysteine
HHcy: hyperhomocysteinaemia
MS: methionine synthase
MTHFR: methylene-tetrahydrofolate reductase
Q: quantitative
RT: real-time
sarcosine dehydrogenase: (SARDH)
tHcy: total plasma homocysteine
